# Retrospective Evaluation of the Association Between Mechanical Power and Outcome in Mechanically Ventilated Dogs from a Multicenter Database (2019–2025): 76 Cases

**DOI:** 10.1111/vec.70159

**Published:** 2026-09-20

**Authors:** Tereza Stastny, Robert H. Markowitz, Dana J. Caldwell, Jiwoong Her, Rachel C. Clarkin‐Breslin, Carolina S. Graham, Jeong E. Park, Deborah C. Silverstein

**Affiliations:** ^1^ University of Pennsylvania School of Veterinary Medicine Philadelphia Pennsylvania USA; ^2^ Shannon Scientific Philadelphia Pennsylvania USA; ^3^ Arizona Veterinary Emergency and Critical Care Center Gilbert Arizona USA; ^4^ North Carolina State University College of Veterinary Medicine Raleigh North Carolina USA; ^5^ Ohio State University College of Veterinary Medicine Columbus Ohio USA; ^6^ Department of Internal Medicine, Cardiovascular Medicine University of California, Davis Davis California USA

**Keywords:** canine, lung injury, mechanical ventilation, respiratory rate

## Abstract

**Objective:**

To assess the association between mechanical power (MP) and survival in mechanically ventilated dogs with primary pulmonary parenchymal disease and to compare its prognostic performance against individual ventilator parameters and oxygenation indices.

**Design:**

Retrospective, multicenter observational cohort study.

**Setting:**

Three veterinary medical teaching institutions and one private veterinary referral center.

**Animals:**

Seventy‐six client‐owned dogs mechanically ventilated for primary pulmonary parenchymal disease between March 2019 and March 2025.

**Interventions:**

None.

**Methods:**

MP (J/min/kg) was calculated using a simplified equation for pressure‐controlled (PC) ventilation: 0.098 × respiratory rate × tidal volume × (dynamic driving pressure + positive end‐expiratory pressure). Median values were calculated per patient for each of the first 3 ventilation days. Multivariate logistic regression was used to assess associations between MP, its components, and survival. Area under the receiver operating characteristic curve was calculated to evaluate predictive performance.

**Results:**

Survivors had significantly lower MP values than nonsurvivors across all 3 initial days of ventilation (Day 1: 0.30 vs. 0.57 J/min/kg; Day 2: 0.33 vs. 0.55 J/min/kg; Day 3: 0.31 vs. 0.47 J/min/kg; *p* = 0.008 to <0.001). A 0.05‐J/min/kg increase in MP was associated with a 19%, 26%, and 34% greater likelihood of mortality on Days 1, 2, and 3, respectively, with good discriminative performance (area under the receiver operating characteristic curve = 0.80, 0.84, and 0.84). Dynamic variables (respiratory rate, dynamic driving pressure, peak inspiratory pressure) were more strongly associated with survival than static variables (tidal volume, positive end‐expiratory pressure).

**Conclusions:**

MP is associated with outcome in dogs undergoing mechanical ventilation and demonstrates stronger predictive value than its individual components. These findings align with human data and support considering MP during ventilator management. Further studies are needed to define thresholds and evaluate whether reducing MP improves outcomes.

**Abbreviations**
ABG: arterial blood gas
ARDS: acute respiratory distress syndrome
AUC: area under the receiver operating characteristic curve
IQR: interquartile range
MP: mechanical power
PC: pressure‐controlled
PEEP: positive end‐expiratory pressure
PF: PaO_2_:FiO_2_ ratio
PIP: peak inspiratory pressure
RR: respiratory rate
TV: tidal volume
VILI: ventilator‐induced lung injury
Δ*P*_insp_
: dynamic driving pressure (PIP − PEEP)

## 1 Introduction

Mechanical ventilation is a life‐saving support measure for patients with severe pulmonary disease; however, it can result in additional lung damage, known as ventilator‐induced lung injury (VILI) [1, 2]. Since the 2000 publication of a landmark ARDSNet trial, which demonstrated that low tidal volume (TV) ventilation reduced mortality and increased ventilator‐free days, lung‐protective guidelines have predominantly focused on limiting static parameters, such as TV and plateau pressure [1, 3, 4]. Static measures, however, may not fully capture the potential for lung injury. Recent studies in both animals and people emphasize the importance of monitoring dynamic parameters, which reflect changes during each tidal cycle and provide a more comprehensive understanding of VILI [1, 5, 6, 7]. Consequently, there has been a recent shift toward incorporating dynamic measures alongside static measures to better assess and mitigate VILI.

In 2016, Gattinoni et al. introduced the concept of mechanical power (MP) as a metric to represent the energy delivered by the ventilator to the entire respiratory system per unit time, combining static and dynamic factors, both of which contribute to VILI [6]. Based on the first law of thermodynamics, which states that energy cannot be created or destroyed, only transformed, the energy transferred from the ventilator to the lungs per breath is known as “mechanical energy” [1, 7, 8]. This energy, when dissipated within lung tissues, can produce heat, inflammation, and structural damage to cells and the extracellular matrix [7]. Mechanical energy per breath is calculated as the area within the pressure–volume loop and, when multiplied by respiratory rate (RR), produces the total MP delivered over time [1, 6]:

$$\mathrm{MP}=\mathrm{RR}\times \left(V_{t}\times \left(\frac{V_{t}}{2C}+\frac{V_{t}}{T_{i}}R+\mathrm{PEEP}\right)\right),$$

where *C* = compliance; PEEP = positive‐end expiratory pressure; *R* = resistance; RR = respiratory rate (/min); *T*_i_ = inspiratory time; and *V*_t_ = tidal volume.

The complexity of this calculation, along with its requirement for volume‐controlled ventilation with constant flow rates, reduces its clinical use [1, 9]. However, simplified MP equations now enable calculations in various ventilation modes using routinely available data. Becher et al. introduced a simplified equation applicable to pressure‐controlled (PC) ventilation [9]:

$$\mathrm{MP}=0.098\times \mathrm{RR}\times V_{t}\times \left(\Delta P_{\mathrm{insp}}+\mathrm{PEEP}\right),$$

where PIP = peak inspiratory pressure; Δ*P*_insp_ = PIP − PEEP; and 0.098 = unit conversion factor.

Although Δ*P*_insp_ + PEEP is mathematically equivalent to PIP, the components are presented separately to emphasize that both dynamic (Δ*P*_insp_) and static (PEEP) pressures contribute to total MP [9].

Recent studies in neonatal, pediatric, and adult intensive care populations have shown that increased MP is associated with prolonged mechanical ventilation and increased mortality [10, 11, 12, 13, 14, 15, 16]. Although published MP thresholds associated with increased mortality in adults range from 12 to 22 J/min [11, 12, 17], a universally accepted safe limit has not been established. Despite growing evidence of its clinical relevance in human medicine, MP has not been evaluated in veterinary patients.

The primary objective of this study was to determine whether MP, calculated using the simplified equation by Becher et al., is associated with survival to discharge in dogs mechanically ventilated using pressure control modes. The secondary objective was to evaluate which ventilator parameters within the MP equation (e.g., RR, PEEP, PIP, Δ*P*_insp_) are most predictive of survival, and whether static variables (e.g., TV, PEEP) or dynamic variables (PIP, Δ*P*_insp_) carry more prognostic weight. It was hypothesized that lower MP would correlate with increased survival and that MP, as a composite variable, would outperform its individual components.

## 2 Materials and Methods

Dogs mechanically ventilated for primary pulmonary parenchymal disease between March 2019 and March 2025 were identified from a multicenter mechanical ventilation database containing cases from the Matthew J. Ryan Veterinary Hospital, University of Pennsylvania School of Veterinary Medicine (Philadelphia, PA, USA); The Ohio State University Veterinary Medical Center (Columbus, OH, USA); the Veterinary Hospital of the North Carolina State University College of Veterinary Medicine (Raleigh, NC, USA); and Arizona Veterinary Emergency & Critical Care Center (Gilbert, AZ, USA; Peoria, AZ, USA). The database was accessed in March 2025. Most dogs included in this study have also been included in other publications derived from the same multicenter database [18, 19, 20]. Exclusion criteria were patients not ventilated in PC modes, dogs ventilated for nonprimary pulmonary parenchymal disease, and those with incomplete data collection sheets. Additionally, patients euthanized solely for financial reasons were excluded from the survival analysis.

Records were reviewed, and the following respiratory mechanics and ventilatory parameters were recorded: peak inspiratory pressure (PIP, cm H_2_O), positive end‐expiratory pressure (PEEP, cm H_2_O), exhaled tidal volume (Tve, mL), patient respiratory rate (pRR), inspiratory pressure (Δ*P*_insp_; PIP − PEEP, cm H_2_O), and FiO_2_. Ventilatory parameters were recorded every 4–6 h daily, corresponding with the times arterial blood gas (ABG) samples were obtained. ABG parameters recorded included pH, PaO_2_, and PaCO_2_. The PaO_2_:FiO_2_ (PF) ratios were calculated from ABG data and recorded FiO_2_ values. Non‐temperature‐corrected values were used for the calculation of PF ratios. All institutions used commercially available ventilators^1^^,^ 2, which were routinely calibrated.

MP was calculated using the simplified equation for PC ventilation described by Becker et al.:

$$MP_{\mathrm{pc}}=0.098\times \mathrm{pRR}\times \mathrm{TV}\times \left(\Delta P_{\mathrm{insp}}+\mathrm{PEEP}\right),$$

where PC refers to pressure‐controlled ventilation, Δ*P*_insp_ is the change in airway pressure during inspiration (PIP − PEEP, cm H_2_O), pRR is the patient's respiratory rate (/min), TV is tidal volume (L), and 0.098 is a conversion factor yielding units in J/min [9]. To allow for comparisons across patients of different sizes, MP_pc_ was indexed to body weight (kg), resulting in units of J/min/kg [11].

Additional clinical data retrieved from patient records included signalment, body weight (kg), diagnosis, indication for mechanical ventilation, ventilation mode, duration of mechanical ventilation, and survival to discharge.

### 2.1 Statistical Analysis

Statistical analyses were performed using statistical software^3^^,^ 4. The assumption of normality was assessed by examining *QQ* plots, histograms, and skewness for each variable. Variables with nonnormal distributions were summarized descriptively using median and interquartile range (IQR). The association between ventilator parameters and survival outcomes was evaluated during the first 3 days of mechanical ventilation. For each patient, all available ventilator and blood gas measurements within each 24‐h ventilation period (Day 1, Day 2, and Day 3) were aggregated by calculating the median value for each parameter, yielding a single daily median value per parameter per patient. These daily medians were then pooled across all days, resulting in a dataset containing up to three observations per patient, each representing the median parameter value for a given ventilation day. Key parameters included MP, PF ratio, RR, PEEP, PIP, Δ*P*_insp_, and TV. For each parameter, a multivariate logistic regression model was constructed using the daily median, adjusting for age group, weight, and sex as covariates, with survival status (0 = nonsurvivor, 1 = survivor) as the outcome. Odds ratios were adjusted based on clinically relevant increments: MP per 0.05‐J/min/kg increase, PF ratio per 50‐unit increase, Δ*P*_insp_ per 1‐cm H_2_O increase, RR per 1‐/min increase, PEEP and PIP each per 1‐cm H_2_O increase, and TV per 1‐mL/kg increase. Statistical significance was evaluated using the Wald test, and *p*‐values <0.05 were considered significant.

The discriminative performance of each model was assessed by calculating the area under the receiver operating characteristic curve (AUC) based on model‐predicted probabilities. Ninety‐five percent confidence intervals were derived using nonparametric bootstrapping with 1000 replicates. This approach provided an integrated early‐phase evaluation of each ventilator parameter's predictive value for survival, accounting for variability within patients and across ventilation days.

## 3 Results

A total of 162 dogs receiving mechanical ventilation between March 2019 and March 2025 were included in the database. Seventy‐six dogs met the inclusion criteria. The remaining dogs were excluded due to ventilation in non‐PC modes (68/162 [42.0%]), missing data necessary for MP calculation (13/162 [8.0%]), or euthanasia solely for financial reasons (5/162 [3.1%]).

Of the 76 patients included in the study, 30 (39.5%) were neutered females, eight (10.5%) were intact females, 28 (36.8%) were neutered males, and 10 (13.2%) were intact males. Fourteen breeds were represented in this population, with the most common being mixed‐breed dogs (26/76 [34.2%]), followed by French Bulldog (11 [14.5%]), Bulldog (8 [10.5%]), Boston Terrier (5 [6.6%]), Pug (5 [6.6%]), Shih Tzu (4 [5.3%]), Cavalier King Charles Spaniel (3 [3.9%]), and Boxer (2 [2.6%]). Twelve other breeds (16.0%), each with one patient, made up the remainder of the study population. The median weight of all dogs was 10.7 kg (IQR: 8.4–31 kg), and the median age was 8 years (IQR: 2.3–10 years).

The most common indications for mechanical ventilation are shown in Table 1. The median duration of mechanical ventilation was 2 days for both survivors and nonsurvivors (IQR: 2–4 days for survivors, 1–2 days for nonsurvivors; *p* < 0.05). In this cohort, 30 of 76 patients (39%) survived to discharge, while 46 (61%) either died or were euthanized. Specifically, 14 (18%) were euthanized and 32 (42%) died.

**TABLE 1:** Indication for mechanical ventilation in 76 dogs with primary pulmonary disease at three university and one private practices between March 2019 and March 2025.

Indication	Frequency (*N* = 76)	Percent of population	Survival (%) (*n*/*N*)
Pneumonia	39	51.3%	38.5% (15/39)
ARDS	12	15.8%	16.7% (2/12)
Pulmonary contusions	5	6.6%	60.0% (3/5)
Cardiogenic pulmonary edema	5	6.6%	80.0% (4/5)
Noncardiogenic pulmonary edema	3	3.9%	66.7% (2/3)
Submersion injury	2	2.6%	0.0% (0/2)
Other disease	10	13.2%	40.0% (4/10)

Abbreviation: ARDS, acute respiratory distress syndrome.

Respiratory mechanics, ventilator settings, and oxygenation parameters over the first 3 days of mechanical ventilation are detailed by day in Table 2, which compares survivors and nonsurvivors. Across Days 1–3, survivors had significantly lower MP (0.30–0.33 [IQR: 0.23–0.48] J/min/kg) compared with nonsurvivors (0.47–0.57 [IQR: 0.34–0.84] J/min/kg), higher PF ratios (227.9–275 [IQR: 150.07–474.73] vs. 123.05–195.2 [IQR: 83.03–306.00]), and lower Δ*P*_insp_ (7.80–9.15 [IQR: 5.35–10.45] cm H_2_O vs. 9.10–10.50 [IQR: 7.53–13.00] cm H_2_O). Survivors also demonstrated lower PEEP settings (5.00–5.25 [IQR: 4.00–8.00] cm H_2_O) than nonsurvivors (6.20–6.50 [IQR: 5.00–8.57] cm H_2_O), lower PIP (13.70–14.10 [IQR: 12.00–17.00] cm H_2_O vs. 16.00–18.00 [IQR: 13.50–21.00] cm H_2_O), reduced RR (28.00–31.00 [IQR: 22.00–33.00]/min vs. 36.00–40.00 [IQR: 30.00–60.00]/min), and lower TV (7.30–8.00 [IQR: 6.50–10.00] mL/kg) compared with nonsurvivors (8.70–9.00 [IQR: 7.30–10.60] mL/kg).

**TABLE 2:** Respiratory mechanics, ventilator settings, and oxygenation parameters by survival group and ventilation day in 76 dogs with primary pulmonary disease at three university and one private practices between March 2019 and March 2025.

Day of ventilation	Parameter	Survivor median (IQR)	Nonsurvivor median (IQR)	Adjusted *p*‐value
Day 1 (*n* = 76)				
	Mechanical power (J/min/kg)	0.30 (0.24–0.47)	0.57 (0.39–0.84)	<0.001^***^
	PF ratio	227.90 (150.07–391.02)	123.05 (83.03–226.78)	<0.001^***^
	Δ*P*_insp_ (cm H_2_O)	9.15 (7.05–10.45)	10.50 (7.53–13.00)	0.002^**^
	Tidal volume (mL/kg)	8.00 (6.85–10.00)	8.05 (6.85–9.38)	0.043^*^
	Respiratory rate (/min)	28.00 (22.00–35.00)	40.00 (30.00–57.25)	<0.001^***^
	PEEP (cm H_2_O)	5.00 (3.85–7.00)	7.00 (5.43–8.57)	<0.001^***^
	PIP (cm H_2_O)	14.00 (12.00–17.00)	18.00 (14.15–21.00)	<0.001^***^
Day 2 (*n* = 48)				
	Mechanical power (J/min/kg)	0.33 (0.23–0.48)	0.55 (0.37–0.78)	<0.001^***^
	PF ratio	275.00 (182.85–474.73)	195.20 (113.90–301.85)	<0.001^***^
	Δ*P*_insp_ (cm H_2_O)	8.95 (7.00–10.10)	9.20 (7.05–11.35)	0.211
	Tidal volume (mL/kg)	7.85 (7.00–9.40)	9.00 (7.60–10.60)	0.565
	Respiratory rate (/min)	28.00 (22.00–33.00)	40.00 (30.00–60.00)	<0.001^***^
	PEEP (cm H_2_O)	5.00 (4.00–8.00)	6.20 (5.00–8.50)	0.010^**^
	PIP (cm H_2_O)	14.10 (13.00–16.88)	16.00 (13.50–19.00)	0.008^**^
Day 3 (*n* = 21)				
	Mechanical power (J/min/kg)	0.31 (0.25–0.41)	0.47 (0.34–0.62)	0.008^**^
	PF ratio	245.60 (144.25–325.72)	189.60 (105.40–306.00)	0.034^*^
	Δ*P*_insp_ (cm H_2_O)	7.80 (5.35–9.00)	9.10 (8.20–10.80)	0.005^**^
	Tidal volume (mL/kg)	7.30 (6.50–8.85)	8.70 (7.30–9.70)	0.211
	Respiratory rate (/min)	31.00 (26.00–32.00)	36.00 (30.00–59.00)	0.010^*^
	PEEP (cm H_2_O)	5.25 (4.80–8.00)	6.50 (5.70–8.10)	0.154
	PIP (cm H_2_O)	13.70 (12.00–15.00)	16.00 (15.00–19.00)	0.004^**^

*Note*: Adjusted *p*‐values from logistic regression models controlling for age, weight, and sex. *n* = the number of dogs remaining on Days 1, 2, and 3 (reflecting attrition due to euthanasia or death). Respiratory rate denotes the patient's respiratory rate.

Abbreviations: IQR, interquartile range; PEEP, positive end‐expiratory pressure; PF ratio, PaO_2_:FiO_2_; PIP, peak inspiratory pressure; Δ*P*_insp_, driving pressure calculated as PIP − PEEP.

*p < 0.05.

**p < 0.01.

***p < 0.001.

MP demonstrated consistent predictive accuracy across Days 1–3 (AUC = 0.80–0.83; *p* < 0.001). On Days 1 and 2, MP and RR demonstrated comparable predictive performance (AUC = 0.80 and 0.82 on Day 1; 0.84 and 0.85 on Day 2, respectively; all *p* < 0.001), Table 3. By Day 3, MP remained predictive (AUC = 0.84; *p* = 0.008), while RR showed a slight decline (AUC = 0.81; *p* = 0.010). The PF ratio demonstrated moderate predictive ability across all days (AUC = 0.72–0.79; *p* ≤ 0.034), and Δ*P*_insp_ demonstrated moderate predictive ability on Days 1 and 3 (AUC = 0.74 and 0.79; *p* = 0.002 and 0.019, respectively), but not on Day 2. PIP and PEEP showed AUCs ranging from 0.69 to 0.82, with significance observed on Days 1 and 3. TV consistently had the lowest discriminative performance (AUC = 0.66–0.71), reaching statistical significance only on Day 1 (*p* = 0.043).

**TABLE 3:** Predictive performance of respiratory mechanics, ventilator settings, and oxygenation parameters for the outcome of survival over the first 3 days of mechanical ventilation, including adjusted OR, AUC, sensitivity, specificity, PPV, and NPV.

Ventilator parameter	Ventilation day	Adjusted odds ratio (95% CI)	Adjusted *p*‐value	AUC (95% CI)	Sensitivity	Specificity	PPV	NPV
Mechanical power (J/min/kg)	Day 1	0.83 (0.74–0.89)	<0.001	0.80 (0.74–0.87)	0.63	0.84	0.67	0.81
Mechanical power (J/min/kg)	Day 2	0.74 (0.65–0.84)	<0.001	0.84 (0.78–0.91)	0.67	0.80	0.71	0.77
Mechanical power (J/min/kg)	Day 3	0.66 (0.50–0.86)	0.008	0.84 (0.72–0.96)	0.94	0.72	0.83	0.92
P/F ratio	Day 1	1.25 (1.12–1.43)	<0.001	0.76 (0.68–0.84)	0.49	0.89	0.68	0.78
P/F ratio	Day 2	1.37 (1.17–1.60)	<0.001	0.79 (0.71–0.87)	0.59	0.85	0.74	0.74
P/F ratio	Day 3	1.43 (1.06–1.95)	0.034	0.72 (0.58–0.86)	0.52	0.52	0.73	0.81
Δ Pressure (cm H_2_O)	Day 1	0.84 (0.75–0.95)	0.002	0.74 (0.67–0.81)	0.39	0.82	0.53	0.72
Δ Pressure (cm H_2_O)	Day 2	0.92 (0.82–1.04)	0.211	0.70 (0.62–0.79)	0.60	0.66	0.56	0.69
Δ Pressure (cm H_2_O)	Day 3	0.59 (0.42–0.82)	0.005	0.79 (0.68–0.91)	0.78	0.80	0.74	0.65
Tidal volume (mL/kg)	Day 1	1.15 (1.01–1.30)	0.043	0.71 (0.64–0.79)	0.23	0.85	0.44	0.68
Tidal volume (mL/kg)	Day 2	0.96 (0.80–1.13)	0.565	0.70 (0.62–0.79)	0.59	0.63	0.54	0.68
Tidal volume (mL/kg)	Day 3	0.83 (0.62–1.11)	0.211	0.66 (0.52–0.80)	0.78	0.32	0.62	0.59
Respiratory rate (breaths/min)	Day 1	0.72 (0.61–0.86)	<0.001	0.82 (0.76–0.88)	0.65	0.85	0.69	0.82
Respiratory rate (breaths/min)	Day 2	0.53 (0.40–0.69)	<0.001	0.85 (0.78–0.91)	0.74	0.77	0.70	0.83
Respiratory rate (breaths/min)	Day 3	0.59 (0.40–0.87)	0.030	0.81 (0.69–0.95)	0.97	0.64	0.80	0.94
PEEP (cm H_2_O)	Day 1	0.68 (0.57–0.81)	<0.001	0.79 (0.72–0.85)	0.42	0.84	0.56	0.73
PEEP (cm H_2_O)	Day 2	0.80 (0.69–0.94)	0.030	0.75 (0.67–0.83)	0.62	0.68	0.59	0.71
PEEP (cm H_2_O)	Day 3	0.84 (0.67–1.06)	0.154	0.69 (0.54–0.83)	0.86	0.32	0.65	0.62
Peak inspiratory pressure (cm H_2_O)	Day 1	0.79 (0.72–0.86)	<0.001	0.79 (0.75–0.86)	0.45	0.85	0.60	0.75
Peak inspiratory pressure (cm H_2_O)	Day 2	0.84 (0.74–0.95)	0.008	0.76 (0.68–0.84)	0.64	0.71	0.62	0.73
Peak inspiratory pressure (cm H_2_O)	Day 3	0.73 (0.59–0.89)	0.004	0.82 (0.72–0.92)	0.81	0.64	0.76	0.78

*Note*: Adjusted odds ratios (95%) are interpreted per unit increase: mechanical power (0.5 J/min/kg), P/F ratio (20 mm Hg), Δ pressure (1 cm H_2_O), tidal volume (1 mL/kg), respiratory rate (5 breaths/min), PEEP (1 cm H_2_O), and peak inspiratory pressure (1 cm H_2_O).

Regression models found that a 0.05‐unit increase in MP was associated with a 19%, 26%, and 34% higher likelihood of mortality on Days 1, 2, and 3 of mechanical ventilation, respectively (odds ratio: 0.81 [95% confidence interval: 0.74–0.89], 0.74 [0.65–0.84], and 0.66 [0.50–0.88]; *p* ≤ 0.008).

## 4 Discussion

The results of the current study support the hypothesis that lower MP, normalized to body weight, is associated with improved survival rates in mechanically ventilated dogs. A 0.05‐unit increase in MP was associated with greater likelihood of mortality on Days 1, 2, and 3 of mechanical ventilation, suggesting that MP may serve as a useful prognostic indicator and risk stratification tool. Among the ventilatory parameters contributing to MP, none independently demonstrated the same predictive strength for survival, highlighting MP's value as a global composite measure. However, dynamic parameters (RR, Δ*P*_insp_, PIP) had a greater impact on survival compared with static parameters (PEEP, TV), suggesting that ventilatory strategies aimed at minimizing RR, Δ*P*_insp_, and PIP may enhance patient outcomes.

MP consistently predicted mortality with increasing accuracy over the first 72 h of ventilation (AUC = 0.81, 0.84, and 0.84 on Days 1, 2, and 3, respectively). These findings align with human studies, where MP has been associated with mortality and demonstrated good prognostic value both in populations with acute respiratory distress syndrome (ARDS) (AUC = 0.75–0.80) [12, 14, 17, 21, 22] and those without ARDS (AUC = 0.69–0.73) [23, 24].

RR demonstrated similar predictive performance to MP early on, suggesting it may serve as a simpler discriminator at initial stages of mechanical ventilation, although prospectively controlling for confounding factors such as sedation, PaCO_2_ targets, neuromuscular blockade, and patient size would be needed. Additionally, although both MP and RR showed strong predictive ability on Day 1, early values may reflect transient factors such as inadequate sedation, atelectasis, suboptimal ventilator settings, or asynchrony, which limits their standalone interpretability. In contrast, persistently elevated respiratory mechanics beyond the first 24 h are more likely to reflect progressive lung injury. Notably, all but seven nonsurvivors in this cohort died within the first 72 h. To minimize survivor bias, MP values obtained beyond 72 h were excluded from analysis.

The association between higher MP and nonsurvival was expected, as MP increases with higher TV, PEEP, RR, and Δ pressure [6]. This may help explain why MP demonstrated the highest cumulative AUC (0.83) across all time points, while most of its individual components, aside from RR, fell within the moderately predictive range, with average AUCs between 0.69 and 0.76. Differentiating which variables reflect underlying disease severity from those that directly contribute to VILI and are modifiable by ventilation strategies may have important clinical implications. For example, patients with severe hypoxemia often require higher PEEP to improve oxygenation [25, 26], indicating that PEEP may act more as a marker of disease severity than a direct cause of harm. In contrast, higher TV and Δ*P*_insp_ have been directly linked to VILI [3, 27] and could represent modifiable targets for lung‐protective strategies, such as low TV ventilation and permissive hypercapnia [3, 28].

When distinguishing between static (TV, PEEP) and dynamic (RR, PIP, Δ pressure) variables, only dynamic variables were significantly different between survivors and nonsurvivors across all time points. Both TV and PEEP lost significance by Days 2 and 3, respectively. These findings support the theory that cyclical mechanical stress, induced by repetitive lung inflation and deflation, may be more injurious than static loading [29, 30, 31, 32]. This is consistent with human and animal studies demonstrating an association between greater magnitude dynamic parameters and worse outcomes [33, 34, 35, 36]. A high RR may be intrinsically harmful due to the higher cumulative mechanical burden it imposes, resulting in increased energy delivered to the lungs and a heightened risk of VILI [36, 37, 38]. The fact that RR achieved the highest AUC of any individual parameter highlights its potential clinical importance. If validated in prospective veterinary trials, these findings would support targeting low Δ*P*_insp_, PIP, and RR as key elements of lung‐protective ventilation.

TV showed the poorest predictive value, losing significance by Day 2. This likely reflects the narrow range used (median: 8.15 mL/kg; IQR: 7.02–9.66) due to widespread adoption of lung‐protective strategies. This finding therefore does not diminish TV's role in VILI, but rather suggests practices are already aligned with evidence from human medicine showing improved outcomes with lower TVs [3, 39].

MP outperformed the PF ratio in predicting mortality, with higher AUC values across all time points. The PF ratio remains the most widely used metric for assessing pulmonary function and stratifying acute hypoxemic respiratory failure in both human and veterinary medicine [40, 41]; however, its limitations are increasingly recognized [42, 43, 44]. Guidelines, including human ARDS definitions and PEEP titration tables, often use PF ratio thresholds to guide respiratory support [40], but the PF ratio primarily reflects the severity of hypoxemia rather than the mechanical forces being imposed on the lungs during ventilation [43]. In contrast, MP integrates key ventilator settings such as TV, Δ*P*_insp_, and PEEP, which directly influence the risk of VILI. This may explain why MP demonstrated superior predictive performance. These findings suggest that incorporating direct measures of respiratory mechanics, whether through MP or comparable integrative metrics, may offer a more accurate assessment of VILI risk, enhance lung‐protective ventilation strategies, and improve outcome prediction in mechanically ventilated critically ill patients.

Veterinary patients differ meaningfully from people in terms of size, conformation, thoracic compliance, and disease phenotype, factors that complicate the direct extrapolation of MP thresholds across species. To account for differences in body size, we normalized MP to body weight, a method also employed in pediatric human medicine. When comparing ventilated children with primary pulmonary parenchymal disease with this canine cohort, similar patterns emerged. Survivors demonstrated lower normalized MP values (0.33 [IQR: 0.27–0.46] in children vs. 0.30 [IQR: 0.24–0.47] in dogs), whereas nonsurvivors had substantially higher values (0.49 [IQR: 0.37–0.57] in children vs. 0.57 [IQR: 0.39–0.84] in dogs) [45]. These parallel findings reinforce the association between increased MP and worse outcomes in two more heterogeneous populations. However, important differences remain, particularly in conformation, thoracic mechanics, and disease presentation between human and veterinary medicine, which warrant further investigation. Additionally, nearly 40% of our cohort were brachycephalic breeds, raising the possibility that conformation‐specific pulmonary mechanics may have influenced results. Future studies stratifying MP by brachycephalic versus nonbrachycephalic conformation may help address this potential bias.

This study has several limitations. First, the absence of plateau pressure measurements prevented differentiation between the resistive and elastic components of MP. Second, the analyses were restricted to patients ventilated in PC mode, which reduced the sample size and limited the generalizability of findings to other ventilation modes. Additionally, in some patients switched to PC synchronized intermittent mandatory ventilation during the first 3 days, the MP formula for PC ventilation would slightly underestimate MP for pressure‐supported breaths. Third, cases ventilated for nonprimary pulmonary parenchymal disease were excluded due to low numbers and frequent confounding by concurrent parenchymal disease. While necessary, this exclusion may have limited the ability to establish comparative reference values for MP in healthy lungs. Comorbidities such as rib fractures or obesity, which may alter chest wall compliance, could also have influenced MP values, representing an additional source of variability; these factors were not fully accounted for due to the retrospective nature of the study. Furthermore, variability in the timing and frequency of data collection may have introduced sampling bias, with patients receiving more frequent monitoring potentially being overrepresented. To mitigate this, aggregate median values were used in the analysis; however, prospective, standardized data collection would enhance consistency and reliability. As is common in veterinary critical care, mortality outcomes may have been influenced by nonmedical factors, such as euthanasia due to financial constraints rather than disease severity. To partially account for this, patients whose owners stated that euthanasia was solely for financial reasons were excluded. Additionally, the study did not stratify patients by illness severity using a validated scoring system, limiting adjustment for baseline risk. Although multivariate logistic regression was employed to adjust for known covariates, the retrospective design and lack of standardized severity indices mean that confounders such as differences in disease processes, comorbidities, or baseline health status may have influenced outcomes. Therefore, although MP was associated with mortality, this relationship is not necessarily causal and should be validated in future prospective studies. Finally, most cases included in this study have been reported in other publications arising from the same multicenter mechanical ventilation database addressing different research questions. These publications should not be considered independent cohorts for future meta‐analyses.

MP was a consistent predictor of mortality in mechanically ventilated dogs and outperformed its individual components and oxygenation indices. The cumulative impact of ventilator settings is reflected by MP and may help identify patients at risk for VILI. These findings align with human data and support considering MP in ventilator management. Further studies are needed to define thresholds and evaluate whether reducing MP improves outcomes.

## Author Contributions

**Tereza Stastny**: conceptualization, investigation, writing – original draft, methodology, validation, visualization, writing – review and editing, formal analysis, project administration, data curation, supervision, resources. **Robert H. Markowitz**: conceptualization, formal analysis, writing – review and editing, data curation. **Dana J. Caldwell**: conceptualization, writing – review and editing, data curation. **Jiwoong Her**: writing – original draft, formal analysis, data curation. **Rachel C. Clarkin‐Breslin**: writing – review and editing, methodology, data curation. **Carolina S. Graham**: data curation, writing – review and editing, project administration. **Jeong E. Park**: data curation, project administration, writing – review and editing. **Deborah C. Silverstein**: conceptualization, writing – original draft, writing – review and editing, methodology, supervision, formal analysis.

## Ethics Statement

The authors confirm that the ethical policies of the journal, as noted on the journal's author guidelines page, have been adhered to. No ethical approval was required as this is a retrospective study.

## Conflicts of Interest

The authors declare no conflicts of interest.

## Data Availability

The data that support the findings of this study are available from the corresponding author upon reasonable request.

## Endnotes

1 Servo‐U Ventilator System, Getinge, Gothenburg, Sweden. https://www.getinge.com

2 Servo‐I Ventilator System, Getinge. https://www.getinge.com

3 R, R Core Team.

4 SAS 9.4, Cary, NC.
